# Transfusion Reactions in Paediatric Patients; Hemovigilance Data from a Tertiary Hospital

**DOI:** 10.3390/children12121662

**Published:** 2025-12-08

**Authors:** Fatma Durak, Özlem Tezol

**Affiliations:** 1 Blood Center, Mersin University Hospital, 33110 Mersin, Turkey; 2 Department of Pediatrics, Faculty of Medicine, Mersin University, 33110 Mersin, Turkey; ozlemtezol@mersin.edu.tr

**Keywords:** hemovigilance, paediatric patients, transfusion reactions

## Abstract

**Objective**: This study aimed to define transfusion-related adverse reactions (TRs) observed in paediatric patients at a university hospital in Turkey. **Methods**: The data from the archive of the Mersin University Hospital Blood Centre, spanning the period between August 2017 and August 2024, were subjected to retrospective analysis. The descriptive and clinical characteristics of paediatric patients who received blood transfusions and were recorded using the haemovigilance reporting system were subjected to analysis. The findings were presented in the form of descriptive statistics. **Results**: Over a seven-year period, 34 TRs were reported, yielding an overall incidence of 1.12‰ (95% CI: 0.79–1.55‰; 34/30,265). The reaction rate was 0.84‰ (95% CI: 0.45–1.42‰; 12/14,329) for erythrocyte concentrates, 1.11‰ (95% CI: 0.58–1.92‰; 11/9948) for fresh plasma and 2.04‰ (95% CI: 1.07–3.55‰; 11/5384) for platelet concentrates. The per patient incidence of TRs was 8.81‰ (95% CI: 6.20–12.17‰; 34/3861). A total of 35.3% of TRs were associated with erythrocyte concentrate, 32.4% with fresh plasma and 32.4% with platelet concentrate. The types of TRs were as follows: mild allergic reaction (64.7%), febrile non-haemolytic transfusion reaction (17.6%), anaphylactic reaction (5.9%), transfusion-related dyspnoea (5.9%), acute haemolytic reaction (2.9%) and acute unspecified transfusion reaction (2.9%). No errors were identified in the pre-transfusion process in any of the patients. **Conclusions**: Allergic and febrile non-haemolytic TRs are among the most commonly observed transfusion reactions in paediatric patients. The analysis of these reactions can be enhanced through the implementation of haemovigilance systems. The implementation of robust haemovigilance systems is crucial for the enhancement of preventive and corrective measures.

## 1. Introduction

**Figure 1:**
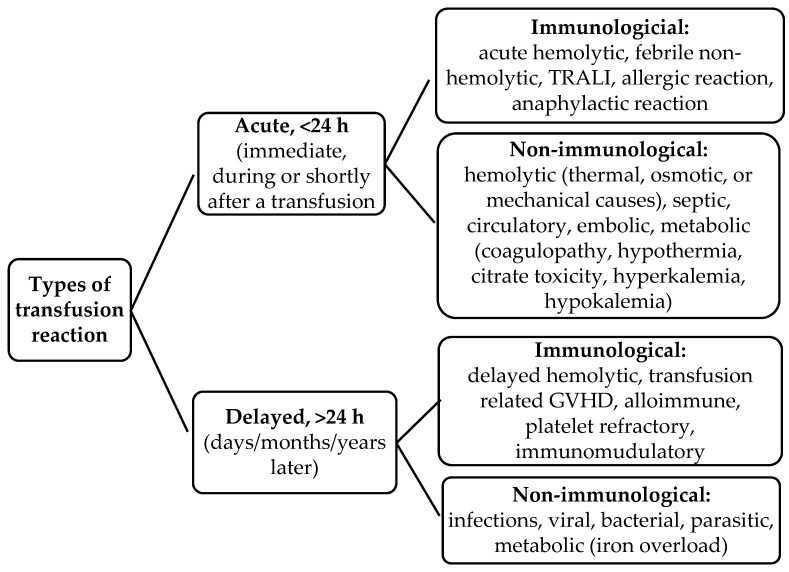
Transfusion-related adverse reactions. TRALI, transfusion-related acute lung injury, GVHD, Graft Versus Host Disease.

Blood transfusion represents a fundamental aspect of contemporary paediatric healthcare. When administered correctly, it has the potential to support saving lives and improve health outcomes. Nevertheless, there are inherent risks associated with blood transfusion, including both infectious and non-infectious adverse effects [1,2]. Transfusion reactions (TRs) are adverse events that occur in patients in relation to the transfusion of blood or blood components. Transfusion-related adverse reactions in patients are classified into two categories: early (acute) and delayed reactions. An acute transfusion reaction (ATR) is defined as a reaction that manifests within the first 24 h following transfusion, typically within the first four hours. Delayed reactions are defined as those that manifest after a period of 24 h following transfusion. Furthermore, adverse reactions can be classified as either ‘immune’ or ‘non-immune’ (Figure 1) [3,4].

The World Health Organization defines haemovigilance as a set of surveillance procedures covering the entire transfusion chain, from the donation and processing of blood and its components to their provision and transfusion to patients and their follow-up [1]. The objective of haemovigilance is to collect and evaluate information pertaining to any unexpected or undesirable events that may arise from the clinical utilisation and collection of blood and blood components. Furthermore, the aim is to prevent the occurrence or recurrence of such incidents. The remit of haemovigilance encompasses all adverse reactions occurring in the context of blood donation or transfusion, as well as all adverse events occurring within the transfusion chain from collection of blood (components) to follow-up of its recipients. Additionally, it incorporates procedures for epidemiological follow-up of blood donors. The principal objective of haemovigilance is to enhance the safety of both recipient and blood donors by preventing the recurrence of adverse reactions and events [1,2,3]. The implementation of effective national and international haemovigilance systems has been demonstrated to have a substantial impact on the enhancement of public health and the quality of patient care. National haemovigilance systems started in France after the FVIII/haemofilia drama in the early 1980s, followed by the UK (SHOT system) and the Netherlands (TRIP system), and spread over the world. In Turkey, a national haemovigilance system was introduced in 2016, and the National Haemovigilance Guideline was drafted with the financial assistance of the European Union [1,2,3,4,5].

The incidence of TRs may vary in relation to the blood or blood component used, the patient group in question and the age of the recipient. The data from haemovigilance systems and single-centre studies indicate that transfused paediatric patients experience TRs more frequently than adults [6]. The number of transfusions of erythrocytes, platelets and plasma administered to neonates and children is increasing, yet the majority of countries’ haemovigilance systems lack the capacity to assess data specific to paediatrics [7]. The number of studies examining the frequency and reaction types of TRs in paediatric patients is limited. It is evident that further studies are required to evaluate TRs experienced by paediatric patients. This will facilitate improvements in reporting practices and reporting systems, as well as enhancing the existing literature on paediatric TRs [8]. The hospital in Mersin Province, located in southern Turkey, is a tertiary university hospital, and Mersin University Hospital Blood Center is inspected and monitored within the framework of Health Quality Standards determined by the Ministry of Health. The haemovigilance system has been operational since 2017 in our hospital. The objective of this study was to ascertain the frequency and nature of TRs in the paediatric population undergoing transfusions at our hospital. The dissemination of haemovigilance experiences may facilitate the enhancement of blood transfusion safety in neonatal and paediatric patients, while also increasing the awareness of paediatricians regarding TRs.

## 2. Materials and Methods

The cross-sectional descriptive study included paediatric patients who developed transfusion-related adverse reactions at our hospital between August 2017 and August 2024. Initially, the total number of paediatric patients (n = 3861) who received blood and blood component transfusions was recorded by looking at the digital archive in our hospital’s statistics unit. Subsequently, the transfusion-related adverse reaction forms (n = 34) in the blood centre archive of our hospital were analysed. The data recorded on the forms were as follows: (i) patient information, including age, sex and blood group; (ii) component information, including whole blood, erythrocyte concentrate, platelet concentrate, fresh plasma and cryoprecipitate; (iii) signs and symptoms, along with laboratory investigations; (iv) transfusion-related adverse reactions, including reaction severity and imputability; (v) transfusion information, including the time of occurrence of the adverse reaction, verification of the patient and component, cross-comparison check and concomitant therapies; (vi) treatment information; and (vii) clinical control of the transfusion process and haemovigilance nurse control: error in the pre-transfusion process, repetition and verification of the patient blood group and cross-comparison. The definitions of blood components, transfusion-related reactions, reaction severity and imputability were based on the National Haemovigilance Guidelines [8].

This study was performed in line with the principles of the Declaration of Helsinki. Ethical permission for the study was obtained from the local ethics committee (4 September 2024/799).

### Statistical Analysis

The statistical software used was SPSS version 21.0 (IBM Corp., Armonk, NY, USA). Categorical variables are presented as numbers and percentages, while numerical variables are presented as the mean ± SD or the median (interquartile range, 25th–75th percentile). The 95% confidence interval (CI) was provided for the incidence data (https://www.openepi.com/PersonTime1/PersonTime1.htm, accessed on 2 October 2024).

## 3. Results

A review of the blood centre records from our hospital revealed that 30,265 units of blood and/or blood components were administered to 3861 paediatric patients between August 2017 and August 2024. The blood and blood components used consisted of 14,329 units of erythrocyte concentrate (47.3%), 9948 units of fresh plasma (32.9%), 4028 units of apheresis platelet concentrate (13.3%, paediatric apheresis platelets/pedipaks for young children and apheresis platelets for older children), 1356 units of pooled platelet concentrate (4.5%), 582 units of cryoprecipitate (1.9%) and 22 units of whole blood (0.07%). A total of 34 TRs were observed. All of these reactions were ATRs that developed within the first 24 h of transfusion. Of the transfusion reactions observed, 32 (94.1%) were first-time reactions, while two (5.9%) were recurrent reactions. No premedication was administered to any patient.

The overall incidence of TR (=ATR incidence) was 1.12‰ (95% CI: 0.79–1.55‰; 34/30,265). The incidence of reactions was 0.84‰ (95% CI: 0.45–1.42‰; 12/14,329) for erythrocyte concentrates, 1.11‰ (95% CI: 0.58–1.92‰; 11/9948) for fresh plasmas and 2.04‰ (95% CI: 1.07–3.55‰; 11/5384) for platelet concentrates [2.48‰ (95% CI: 1.26–4.43‰; 10/4028) in apheresis platelet concentrates and 0.74‰ (95% CI: 0.04–3.64‰; 1/1356) in pool platelet concentrates].

The overall incidence of TRs per patient was 8.81‰ (95% CI: 6.20–12.17‰; 34/3861), comprising erythrocyte concentrate (6.41‰; 95% CI: 3.47–10.9‰; 12/1872), platelet concentrate (11.89‰; 95% CI: 6.25–20.67‰; 11/925) and fresh plasma (10.98‰; 95% CI: 5.77–19.08‰; 11/1002).

The data pertaining to the patients and the blood components are presented in Table 1. The study sample comprised 10 female and 24 male patients, with an age range of 7 days to 17.5 years. The occurrence of TRs was found to be associated with erythrocyte concentrates (35.3%), fresh plasma (32.4%) and platelet concentrates (32.4%), with the majority of these transfusions being apheresis (29.4%) or pooled platelet concentrates (2.9%).

The most frequently observed symptoms during the reaction were redness (52.9%), itching (44.1%), rash (41.2%), urticaria (38.2%), fever (20.6%) and restlessness (20.6%). One patient (2.9%) exhibited clinical and laboratory indications of intravascular haemolysis (Table 2).

The type, severity and imputability of TRs are presented in Table 3. The frequency of mild allergic reactions was 64.7%, febrile non-haemolytic transfusion reactions (FNHTRs) were 17.6%, anaphylactic reactions were 5.9%, transfusion-related dyspnoea was 5.9%, acute haemolytic reactions were 2.9% and acute unspecified transfusion reactions were 2.9%. Only mild allergic reactions related to fresh plasma transfusion were observed. Transfusion-related dyspnoea, acute haemolytic reaction and acute unspecified transfusion reaction were found to be associated with the erythrocyte concentrates. Anaphylactic reactions were found to be associated with platelet concentrates. The most prevalent adverse reactions associated with erythrocyte concentrates were FNHTRs, while the most common reactions associated with platelet concentrates were mild allergic reactions. The majority of reactions (88.2%) were classified as non-severe, and the majority of cases (82.4%) were classified as probable. No mortality was recorded over the seven-year period.

In patients who developed TRs, the clinical signs manifested at the median of 45 min (ranging from 5 to 240 min) and 205 mL (ranging from 20 to 2400 mL) of transfusion. In addition to stopping the transfusion, antihistamines (70.6%) and corticosteroids (50.0%) were the most commonly administered medications (Table 3).

TRs were observed in 76.5% (26/34) of cases in inpatient wards, 14.7% (5/34) in the paediatric intensive care unit, 5.9% (2/34) in the neonatal intensive care unit and 2.9% (1/34) in the paediatric emergency department. Of the patients, 61.8% (n = 21) had a haematological–oncological disease and 91.2% (n = 31) had a history of repeated transfusion. No errors were identified in the pre-transfusion process in any of the patients (Table 4).

## 4. Discussion

The present study investigated paediatric TRs observed in a university hospital with a haemovigilance system over a seven-year period. All observed reactions were classified as ATRs. The most prevalent ATRs were mild allergic reactions, with the majority of cases associated with platelet concentrate. FNHTRs were the second most common, with the majority of cases associated with erythrocyte concentrate.

The haemovigilance system data indicate that the incidence of ATR in the paediatric population is 1.35‰ in China, 5.38–6.16‰ in the USA, 4.75‰ in Brazil, 8.08‰ in India, 1.70‰ in Pakistan and 2.35‰ in Turkey [8,9,10,11,12,13,14]. The incidence of the condition in transfusion-dependent paediatric patients (those with blood malignancy and thalassaemia) in Italy is 2.63 per 100.000, while in transfused patients in the paediatric intensive care unit in Canada it is 1.59 per 100.000 [15,16]. It is possible that the incidence of the condition may have been influenced by a number of factors, including differences in age groups, races, underlying diseases, the definitions of reactions and the notification and reporting practices associated with ATRs. In the present study, the incidence of ATRs was 1.12‰. Certainly, our single-centre experience may not accurately reflect national experience.

The distribution of ATR frequency according to the transfused blood product may be characterised by a preference for platelet > erythrocyte > fresh plasma or platelet > fresh plasma > erythrocyte or, alternatively, by a tendency towards erythrocyte > platelet > fresh plasma or erythrocyte > fresh plasma > platelet [8,10,13,14]. The distribution of the ATRs observed in our study was found to be platelet > fresh plasma > erythrocyte. It has been documented that paediatric patients have experienced allergic reactions with cryoprecipitate and FNHTRs, as well as TRALI (transfusion-related acute lung injury) with whole blood [12,17]. The administration of cryoprecipitate or whole blood transfusion did not result in the occurrence of ATRs in our study. In previous studies in which the most commonly transfused product was erythrocyte concentrate, as in our study, ATRs developed at a mean of 223 ± 71 mL and a median of 30 min after transfusion [12,14]. In the present study, the median dose and median duration of ATRs were found to be 205 mL and 45 min, respectively.

The per patient ATR event in a paediatric hospital in China between 2015 and 2019 included 0.37‰ with erythrocyte concentrate, 2.98‰ with platelet concentrate and 1.65‰ with fresh plasma [8]. The present study revealed a higher incidence of ATRs per patient in all blood components. The total number of patients transfused, the total number of products transfused, the amount of products transfused per child (which varies with age and body weight) and the distribution of ATR frequencies according to blood components may serve to elucidate the discrepancies observed in the incidence of ATRs per patient.

Allergic TRs represent the most frequently reported type of reaction in paediatric patients. The symptoms may range from mild urticaria and rash to severe angioedema and bronchospasm/respiratory distress [6,14]. The prevalence of allergic TRs in paediatric patients in the USA has been documented to range from 2.7 to 3.2 per 1000 transfusions [9,10]. In Brazil, 77% of paediatric TRs were reported to be allergic, with the majority being associated with platelets [11]. In Japan, the incidence of allergic transfusion reactions in paediatric patients was observed to be four times higher than that of FNHTRs, with the most common association being with platelets [18]. Allergic reactions are the underlying cause of 86.7% of TRs in Chinese children and 58.3% in Pakistani children [8,13]. In the present study, the incidence of allergic TRs was found to be 0.79 per 1000 transfusions. Furthermore, 70.6% of all reactions were of an allergic nature, with 64.7% classified as mild allergic reactions and 5.9% as anaphylactic reactions. Notably, the incidence of allergic reactions was found to be highest in platelet transfusions.

A mild allergic reaction is characterised by the presence of mucocutaneous signs and symptoms that manifest during the transfusion process or within the initial four-hour period. This type of allergic reaction is typically not life-threatening and responds rapidly to symptomatic treatment with antihistamines or steroids [4]. It has been documented that cutaneous manifestations are the most prevalent form of presentation in paediatric cases of allergic TRs [13,19]. The findings of this study indicate that cutaneous symptoms and urticaria are the most common symptoms in children with allergic ATRs. Furthermore, antihistamines and corticosteroids are the most commonly used treatments. An allergic reaction is defined as anaphylactic in nature if it affects the respiratory and/or cardiovascular system in addition to the mucocutaneous system [4]. Guo et al. reported that 141 of the 143 cases of transfusion-related allergic reactions manifested as urticaria/rash, while the remaining two cases presented as anaphylactic shock [8]. Transfusion-associated anaphylactic reactions in paediatric patients are classified as severe reactions and are most commonly associated with platelets, followed by fresh plasma [14,20]. In both cases, the two anaphylactic reactions observed in the course of our study were also classified as severe and associated with platelet transfusion.

A diagnosis of FNHTR is made when a patient presents with a fever (38 °C or above, or a ≥1 °C increase from the pre-transfusion value) or chills occurring during or within four hours after transfusion, in the absence of bacterial contamination or underlying disease. It is hypothesised that cytokines released from residual white blood cells or recipient antibodies against donor antigens are responsible for FNHTRs [4,6]. FNHTRs represent the second most prevalent form of ATRs, following allergic reactions. In the paediatric population, FNHTRs develop with the greatest frequency in the context of erythrocyte transfusions [9,10,13,18]. In our study, the incidence of FNHTRs was 0.20‰, the prevalence was 17.6% (the second most common type of ATR) and the most common blood component associated with FNHTRs was erythrocyte concentrates.

Transfusion-related dyspnoea is defined as respiratory distress occurring within the first 24 h of transfusion and not attributable to TRALI, TACO (transfusion-associated circulatory overload) or an allergic reaction [4]. Previously, the incidence of transfusion-related dyspnoea in paediatric patients was reported to be 0.05 per 1000 transfusions [9]. The incidence in our study was 0.07‰. In a recent study, Çelik and colleagues reported that 9 (4.3%) of 211 ATRs were transfusion-related dyspnoea [14]. A prevalence of 5.9% was observed for transfusion-related dyspnoea among ATRs, which was found to be associated with erythrocyte concentrates. Other pulmonary ATRs, TRALI and TACO have been documented in neonatal, infant and paediatric patients, with a high prevalence of association with erythrocyte concentrates [9,10,20,21,22]. We did not observe any TRALI or TACO reactions over the seven-year period.

An acute haemolytic transfusion reaction is diagnosed on the basis of clinical and laboratory findings of haemolysis occurring within 24 h of transfusion [4]. In the paediatric population, Vossoughi et al. reported an incidence of 0.09‰, while Oakley et al. reported no acute haemolytic transfusion reactions [9,10]. The incidence observed in the present study was 0.03‰. The majority of acute haemolytic transfusion reactions associated with erythrocyte concentrates occur as a result of mistransfusion. Mistransfusion may occur primarily due to misidentification of the specimen or blood sample, although misidentification of the patient may also be a contributing factor [23]. Wrong blood in tube errors can result in ABO mistransfusions with an estimated frequency range from 4.3 to 5.8 per 10,000 samples, and the relative frequency of wrong-blood-in-tube errors may be higher in paediatric specialties [24,25]. In the present study, the sole instance in which an acute haemolytic reaction was identified was in a case of non-ABO related immune-mediated haemolysis, wherein the transfused erythrocytes and cross-matching control were deemed appropriate. In all instances where an ATR was reported, both the patient and the blood component were confirmed, and no transfusion or transfusion process error was identified. We did not observe any wrong-blood-in-tube errors.

Adverse reactions that do not correspond to any of the pre-defined TRs and cannot be attributed to a cause other than transfusion, but whose relationship with transfusion has not yet been proven, are classified as unidentified TRs [4]. It is estimated that approximately 10% of TRs in children presenting with symptoms such as cough, chest tightness, wheezing, abdominal discomfort or pain, nausea and vomiting, chills, tachycardia, dizziness, tinnitus, body shaking or cyanosis may not be identified. [8,12,14]. In the course of our investigation, a 6-year-old male patient exhibited an unidentified transfusion reaction associated with an erythrocyte concentrate, manifesting as nausea and vomiting.

The overwhelming majority of paediatric ATRs are not severe [12]. In their report, Johns et al. stated that 93% of ATR cases were non-severe, 6% were severe and 1% were life-threatening [23]. In our study, the respective frequencies were 88.2%, 11.8% and 0%. In the paediatric population, the imputability of ATRs is predominantly classified as either possible or probable. Imputability was defined as 39% certain, 18% probable, 36% possible, <1% doubtful and <1% indeterminate by Johns et al. [23]; and 82.6% probable, 15.9% possible and 1.4% unlikely by Ghataliya et al. [12]. In our study, imputability was classified as 82.4% probable, 14.7% possible and 2.9% certain.

Prior research has indicated that between 52 and 81 percent of Turkish children diagnosed with ATRs are male [14,17]. The majority of our patients was also male. The presence of underlying haematological malignancy and a history of transfusion increase the risk of ATRs in paediatric patients [8,12,15,26]. The high prevalence of haematological malignancies and a history of transfusion in our patients who developed ATRs lends further support to this hypothesis. It is possible that patients who develop ATRs may have received transfusion-associated medication, which could potentially affect the accuracy of the diagnosis [12]. Adverse reactions were not associated with these treatments in any of our patients who received concomitant medications. The majority of ATRs in paediatric patients, as in our patients, are treated symptomatically, with a full recovery being achieved in the majority of cases [27].

The most significant limitation of this study is its single-centre and retrospective design. However, the fact that the same haemovigilance nurses have been employed at our blood centre since the introduction of the active haemovigilance system at our hospital is a positive factor in terms of the reliability of the data. A second limitation is that the buffy coat depletion, leukoreduction or irradiation properties of blood products could not be detailed due to the small sample size. A further limitation of the study is that the underlying diseases of patients with ATRs were not analysed in detail. While acute non-immunological or delayed TRs were not observed in this study, it is important to note that paediatric patients may experience a range of TRs, including hypotensive, alloimmune, late haemolytic, infectious and metabolic reactions, as well as transfusion-related graft-versus-host disease [6,12,17], and TRs that developed in the days and months after transfused patients were discharged from our hospital may have been unrecorded. It should also be kept in mind that there may be TRs that have not been reported to the transfusion and blood centres of hospitals [24], and retrospective studies as our study will not include these cases. Lastly, since this is a descriptive study, we did not perform a comparative analysis of the TRs between the periods before and after the hemovigilance system was introduced. We also did not perform a time-trend analysis through hemovigilance. Future multi-centre analytical studies aiming at these analyses should be conducted to improve our national guidelines and principles for safe blood transfusion.

## 5. Conclusions

Allergic and febrile non-haemolytic TRs are among the most commonly observed transfusion reactions in paediatric patients. Prevention strategies such as blood component manipulations (leukoreduction, plasma reduction, additive solutions, etc.) and pharmacology and development of innovative products and new therapeutic approaches should be encouraged. The limited data on paediatric TRs hinders our ability to gain a deeper understanding of the pathophysiology and epidemiology of TRs in the paediatric population. Furthermore, it impedes our capacity to define the reactions with greater precision and to develop strategies to reduce these reactions. It is our contention that our findings may prove to be of value in this field of enquiry. It would be beneficial for future studies to examine both classical TRs and post-transfusion sequelae in greater detail within the paediatric population.

## Figures and Tables

**Table 1:** Patient and blood component characteristics.

	n (%)
Age, years (mean ± SD)	10.4 ± 5.2
<5 years	7 (20.6)
5–9 years	6 (17.6)
≥10 years	21 (61.8)
Total	34 (100.0)
Gender	
Male	24 (70.6)
Female	10 (29.4)
Total	34 (100.0)
Blood groups	
A+	10 (29.4)
B+	5 (14.7)
B-	4 (11.8)
AB+	1 (2.9)
O+	14 (41.2)
Total	34 (100.0)
Blood components	
Erythrocyte concentrates	12 (35.3)
Fresh plasma	11 (32.4)
Apheresis platelet concentrate	10 (29.4)
Pooled platelet concentrate	1 (2.9)
Cryoprecipitate	0 (0)
Whole blood	0 (0)
Total	34 (100.0)
Blood component groups	
A+	10 (29.4)
B+	5 (14.7)
B-	4 (11.8)
AB+	1 (2.9)
O+	14 (41.2)
Total	34 (100.0)
Erythrocyte concentrates	12 (100.0)
Compatible cross-matched, buffy-coat depleted and leuko-reduced	11 (91.7)
Compatible cross-matched, non-leuko-reduced or buffy cot-depleted	1 (8.3)

**Table 2:** Clinical and laboratory characteristics.

	n (%)
Signs and findings	
Rash	18/34 (52.9)
Itching	15/34 (44.1)
Skin eruption	14/34 (41.2)
Urticaria	13/34 (38.2)
Fever	7/34 (20.6)
Discomfort	7/34 (20.6)
Rigours	6/34 (17.6)
Dyspnoea	5/34 (14.7)
Chills	4/34 (11.8)
Nausea, vomiting	2/34 (5.9)
Decrease in oxygen saturation	2/34 (5.9)
Stomach ache	1/34 (2.9)
Cough	1/34 (2.9)
Dysphonia	1/34 (2.9)
Dysphagia	1/34 (2.9)
Fall in haemoglobin, haemoglobinuria, positive Coombs test, increase in serum bilirubin and Lactate dehydrogenase	1/34 (2.9)
Total number of observed symptoms	98 (100.0)

**Table 3:** Acute transfusion reaction characteristics.

	Overall (n = 34)	Erythrocyte Concentrates (n = 12)	Fresh Plasma (n = 11)	Platelet Concentrates (n = 11)
Reaction types				
Mild allergic reactions	22 (64.7)	3 (25.0)	11 (100.0)	8 (72.7)
incidence *	0.73 (0.47–1.08)	0.21 (0.05–0.57)	1.11 (0.58–1.92)	1.49 (0.69–2.82)
FNHTR	6 (17.6)	5 (41.7)	0 (0)	1 (9.1)
incidence *	0.20 (0.08–0.41)	0.35 (0.13–0.77)	-	0.19 (0.009–0.92)
Anaphylactic reactions	2 (5.9)	0 (0)	0 (0)	2 (18.2)
incidence *	0.07 (0.01–0.22)	-	-	0.37 (0.06–1.23)
Transfusion associated dyspnoea	2 (5.9)	2 (16.7)	0 (0)	0 (0)
incidence *	0.07 (0.01–0.22)	0.14 (0.02–0.46)	-	-
Acute haemolytic reaction	1 (2.9)	1 (8.3)	0 (0)	0 (0)
incidence *	0.03 (0.002–0.16)	0.07 (0.004–0.0.34)	-	-
Unknown	1 (2.9)	1 (8.3)	0 (0)	0 (0)
incidence *	0.03 (0.002–0.16)	0.07 (0.004–0.34)	-	-
Reaction severity				
Grade 1/non severe	30 (88.2)	12 (100.0)	10 (90.9)	8 (72.7)
Grade 2/severe	4 (11.8)	0 (0)	1 (9.1)	3 (27.3)
Grade 3/life threatening	0 (0)	0 (0)	0 (0)	0 (0)
Grade 4/fatal	0 (0)	0 (0)	0 (0)	0 (0)
Reaction imputability				
Grade 0/not assessable, unlikely	0 (0)	0 (0)	0 (0)	0 (0)
Grade 1/possible	5 (14.7)	1 (8.3)	1 (9.1)	3 (27.3)
Grade 2/probable	28 (82.4)	10 (83.3)	10 (90.9)	8 (72.7)
Grade 3/certain	1 (2.9)	1 (8.3)	0 (0)	0 (0)
Occurrence				
time, minute of the transfusion	45 (30–65)	104 ± 81	56 ± 32	30 ± 17
dose, mL of the transfusion	205 (150–400)	305 (70–400)	250 (160–400)	200 (200–255)
Concomitant medications				
Antimicrobials and/or IV fluids	7 (17.6)	2 (16.7)	3 (27.3)	2 (18.2)
None	28 (82.4)	10 (83.7)	8 (72.7)	9 (81.8)
Treatment				
Antihistaminic	24 (70.6)	4 (33.3)	10 (90.9)	10 (90.9)
Corticosteroid	17 (50.0)	2 (16.7)	9 (81.8)	6 (54.6)
Antipyretic	6 (17.6)	4 (33.3)	0 (0)	2 (18.2)
Oxygen	2 (5.9)	0 (0)	0 (0)	2 (18.2)
Adrenalin	2 (5.9)	0 (0)	0 (0)	2 (18.2)

FNHTR, febrile non-haemolytic transfusion reaction. Data are number (percentage), median (IQR) or mean ± SD. * transfusion reaction/per 1000 blood product count (95% CI).

**Table 4:** Transfusion reaction frequency by departments and haemovigilance decisions.

	n (%)
Unit where transfusion reaction developed	
Paediatric haematology	12 (35.3)
Paediatric neurology	5 (14.7)
Paediatric intensive care	5 (14.7)
Paediatric infectious diseases	3 (8.8)
Paediatric oncology	3 (8.8)
General paediatrics	2 (5.9)
Neonatology	2 (5.9)
Paediatric gastroenterology	1 (2.9)
Paediatric emergency	1 (2.9)
Total	34 (100.0)
Verification	
of the patient	34 (100)
of the blood product	34 (100)
Repeat ABO grouping and cross-match	
Performed and verified	6 (17.6)
Not performed	28 (82.4)
Total	34 (100.0)
Errors in the pre-transfusion process	0 (0)

## Data Availability

The data that support the findings of this study are available from the corresponding author, [Fatma Durak], upon reasonable request. The data are not publicly available due to ethical reasons.

## Abbreviations

The following abbreviations are used in this manuscript:

TRs	Transfusion-related adverse reactions
ATR	Acute transfusion reaction
CI	Confidence interval
FNHTRs	Febrile non-haemolytic transfusion reactions
TRALI	Transfusion-related acute lung injury
TACO	Transfusion-associated cardiac overload

## References

[1] World Health Organisation A Guide to Establishing a National Haemovigilance System. https://www.who.int/publications/i/item/9789241549844.

[2] Wood E.M., Whitaker B.I., Townsend M., Narayan S. (2024). How we forecast tomorrow’s haemovigilance. Transfus. Clin. Biol..

[3] Turkish Republic Ministry of Health Blood and Blood Products Department Headship (2016). National Hemovigilance Guideline Version 1. https://shgmkanhizmetleridb.saglik.gov.tr/TR-71524/ulusal-hemovijilans-rehberi-versiyon-1-2016.html.

[4] Turkish Republic Ministry of Health Blood and Blood Products Department Headship (2020). National Hemovigilance Guideline Version 2. https://shgmkanhizmetleridb.saglik.gov.tr/TR-71525/ulusal-hemovijilans-rehberi-versiyon-2--2020.html#.

[5] de Vries R.R., Faber J.C., Strengers P.F. (2011). Board of the International Haemovigilance Network. Haemovigilance: An effective tool for improving transfusion practice. Vox Sang..

[6] Sostin N., Hendrickson J.E. (2021). Pediatric Hemovigilance and Adverse Transfusion Reactions. Clin. Lab. Med..

[7] Sapiano M.R.P., Jones J.M., Savinkina A.A., Haass K.A., Berger J.J., Basavaraju S.V. (2020). Supplemental findings of the 2017 National blood collection and utilization survey. Transfusion.

[8] Guo K., Wang X., Zhang H., Wang M., Song S., Ma S. (2021). Transfusion Reactions in Pediatric Patients: An Analysis of 5 Years of Hemovigilance Data From a National Center for Children’s Health in China. Front. Pediatr..

[9] Vossoughi S., Perez G., Whitaker B.I., Fung M.K., Stotler B. (2018). Analysis of pediatric adverse reactions to transfusions. Transfusion.

[10] Oakley F.D., Woods M., Arnold S., Young P.P. (2015). Transfusion reactions in pediatric compared with adult patients: A look at rate, reaction type, and associated products. Transfusion.

[11] Pedrosa A.K., Pinto F.J., Lins L.D., Deus G.M. (2013). Blood transfusion reactions in children: Associated factors. J. Pediatr..

[12] Ghataliya K.J., Kapadia J.D., Desai M.K., Mehariya K., Rathod G., Bhatnagar N., Gajjar M.D. (2017). Transfusion related adverse reactions in pediatric and surgical patients at a ertiary are eaching Hospital in India. Asian J. Transfus. Sci..

[13] Lal J.M., Hasan M., Ali N. (2022). Frequency and types of transfusion reactions in pediatric population: A report from a tertiary care center in Pakistan. Iraqi. J. Hematol..

[14] Kulhas Celik I., Koca Yozgat A., Dibek Misirlioglu E., Bozkaya I.O., Civelek E., Toyran M., Yarali N., Ozbek N.Y. (2021). Frequency and clinical characteristics of allergic transfusion reactions in children. Transfus. Apher. Sci..

[15] De Pascale M.R., Belsito A., Sommese L., Signoriello S., Sorriento A., Vasco M., Schiano C., Fiorito C., Durevole G., Casale M. (2019). Blood transfusions and adverse acute events: A retrospective study from 214 transfusion-dependent pediatric patients comparing transfused blood components by apheresis or by whole blood. Ann. Ist. Super. Sanita..

[16] Gauvin F., Lacroix J., Robillard P., Lapointe H., Hume H. (2006). Acute transfusion reactions in the pediatric intensive care unit. Transfusion.

[17] Duzenli Kar Y., Ozkorucu Yildirgan D., Aygun B., Erdogmus D., Altinkaynak K. (2021). Retrospective evaluation of acute transfusion reactions in a tertiary hospital in Erzurum, Turkey. N. Clin. Istanb..

[18] Yanagisawa R., Tatsuzawa Y., Ono T., Kobayashi J., Tokutake Y., Hidaka E., Sakashita K., Nakamura T. (2019). Analysis of clinical presentations of allergic transfusion reactions and febrile non-haemolytic transfusion reactions in paediatric patients. Vox Sang..

[19] Moncharmont P., Meyer F. (2013). Allergic adverse transfusion reactions in paediatrics, a 3-year study. Transfus. Clin. Biol..

[20] Lieberman L., Petraszko T., Yi Q.L., Hannach B., Skeate R. (2014). Transfusion-related lung injury in children: A case series and review of the literature. Transfusion.

[21] Thalji L., Thum D., Weister T.J., Weber W.V., Stubbs J.R., Kor D.J., Nemergut M.E. (2018). Incidence and epidemiology of perioperative transfusion-related pulmonary complications in pediatric noncardiac surgical patients: A single-center, 5-year experience. Anesth. Analg..

[22] Goel R., Tobian A.A.R., Shaz B.H. (2019). Noninfectious transfusion-associated adverse events and their mitigation strategies. Blood.

[23] Johns C., Bakhtary S., Wu R., Nedelcu E. (2021). A Single-Center Description of Pediatric Transfusion Reactions and Preventable Patient Harm. Hosp. Pediatr..

[24] Dunbar N.M., Kaufman R.M. (2022). WBIT Study Investigators, The Biomedical Excellence for Safer Transfusion (BEST) Collaborative. Factors associated with wrong blood in tube errors: An international case series-The BEST collaborative study. Transfusion.

[25] Varey A., Tinegate H., Robertson J., Watson D., Iqbal A. (2013). Factors predisposing to wrong blood in tube incidents: A year’s experience in the North East of England. Transfus. Med..

[26] Yanagisawa R., Shimodaira S., Sakashita K., Hidaka Y., Kojima S., Nishijima F., Hidaka E., Shiohara M., Nakamura T. (2016). Factors related to allergic transfusion reactions and febrile non haemolytic transfusion reactions in children. Vox Sang..

[27] Li N., Williams L., Zhou Z., Wu Y. (2014). Incidence of acute transfusion reactions to platelets in hospitalized pediatric patients based on the US hemovigilance reporting system. Transfusion.

